# Experiences of sexual violence among women seeking services at a family planning unit in Sweden

**DOI:** 10.1080/03009734.2019.1604587

**Published:** 2019-05-13

**Authors:** Mariella Öberg, Alkistis Skalkidou, Gun Heimer

**Affiliations:** a Department for Women’s and Children’s Health, Uppsala University, Uppsala, Sweden;; b National Centre for Knowledge on Men’s Violence Against Women, Uppsala University, Uppsala, Sweden

**Keywords:** Intimate partner violence, polyvictimization, psychological violence, physical violence, revictimization, sexual violence

## Abstract

**Background:** Experiences of sexual violence among women can lead to ill health and increase the risk of lifetime co-occurrence of violence. Identifying risk factors and victims facilitates development of effective programmes for treatment and prevention of additional violence. The primary aim of this study was to assess the prevalence and correlates of sexual violence experiences among women seeking care at a family planning unit in Sweden. A secondary aim was to examine associations between sexual violence and other types of violence.

**Methods:** Women (*n* = 1226) seeking services at a family planning unit, Uppsala University Hospital, Sweden, answered a questionnaire and were interviewed about experiences of sexual violence. Bivariate associations were examined using the chi-square test.

**Results:** Experiences of sexual violence were reported by 27% of the participants, of which 57% were exposed when they were younger than 18 years old. Women with experiences of sexual violence were more likely to have lower education (*P* = 0.024), were students or without occupation (*P* = 0.037), and were not in a current relationship (*P* < 0.001). Women with experiences of non-partner sexual violence were more likely to have experiences of intimate partner violence (*P* < 0.001).

**Conclusion:** Prevalence of sexual violence was high among the respondents. Many women were young when they were exposed to violence, and lifetime co-occurrence of violence was common among women with experiences of non-partner sexual violence.

## Introduction

Sexual violence against women can be a major threat to the health of millions of girls and women all over the world. Prevalence studies have indicated that 35.6% of women worldwide have experiences of either physical and/or sexual violence (1), and the estimated prevalence of physical and/or sexual violence was 33% among women in European Union countries (2).

In a national prevalence study of lifetime exposure to sexual violence in Sweden, 38% of women reported a history of sexual victimization (3).

Sexual violence has been defined by the World Health Organization (WHO) as: ‘any sexual act, attempt to obtain a sexual act, unwanted sexual comments or advances, or acts to traffic or otherwise directed against a person’s sexuality using coercion, by any person regardless of their relationship to the victim, in any setting, but not limited to home and work’ (4). It can take place in a relationship where a partner perpetrates physical, sexual, and/or psychological violence. The offenders of sexual violence can also be non-partners (e.g. stranger, acquaintance, friend, family member, colleague, military, etc.).

The health consequences of sexual violence against women are well known. Negative effects on health can be short- and/or long-term and include a variety of symptoms and complications such as gynaecological trauma, unintended pregnancy, sexually transmitted infections, sexual dysfunction, and chronic pelvic pain (5–10). Experiences of sexual violence can also have a major impact on mental health, with women reporting depression, posttraumatic stress syndrome, sleeping disorders, and suicidal behaviour (11,12).

According to the ecological model for understanding violence, a number of risk factors have been identified to be associated with experiences of sexual violence on a societal, community, relationship, and individual level such as young age, low education, low socioeconomic status, and exposure to prior abuse (4,13).

Research has shown that lifetime co-occurrence of violence, revictimization, or polyvictimization, among survivors of any type of violence (e.g. physical, sexual, or psychological) is common and can be perpetrated by more than one person during the lifetime of the women exposed (14,15). The patterns of polyvictimization are though not yet fully understood.

Many women with experiences of sexual violence may seek medical help, due to the adverse health consequences described above. The health care system constitutes therefore a significant setting to identify exposed women, and in turn can help to develop effective intervention programmes to prevent further ill health and polyvictimization. A research project aiming to elucidate different aspects of experiences of violence among women seeking family planning services in Uppsala was undertaken during 2005–2006 (16).

The primary aim of the current study was to assess the prevalence and correlates of sexual violence experiences among women seeking care at a family planning unit in Sweden. A secondary aim was to examine associations between sexual violence and other types of violence.

## Materials and methods

### Subjects

The details of the recruiting procedure of the study have been described previously (16). Eligible to participate were Swedish-speaking girls and women from 15 years of age seeking services at the family planning unit in Uppsala’s university hospital during the period of October 2005 to October 2006. Uppsala is the fourth largest city of Sweden with a substantial student population, and the family planning unit services are both for girls and women seeking termination of pregnancy and contraceptive counselling. Information on the study and a self-administered questionnaire were provided to the women at registration. By answering the questionnaire and allowing trained staff to interview them, women gave their consent to participate. Instructions were given to the women to place the questionnaire in a locked box regardless of whether they answered it or not. Confidentiality was assured, and the interviews took place in absence of their partners. If needed, the participants were offered counselling in accordance with current clinical practice.

### Variables

The questions for the interviews consisted of a modified, translated version of the Abuse Assessment Screen (AAS), a validated tool for detection of intimate partner violence and sexual violence (17). A short version of the Norvold Abuse Questionnaire (NorAQ), a tool validated in a Swedish population with questions about psychological, physical, and sexual violence, was used in the self-administered questionnaire (18,19). A number of background questions were also included, for example, age, educational level, occupation, birthplace, and current relationship status. Questions about experiences of sexual violence in the questionnaire were divided into three categories ranging from mild, to moderate and severe:

1. *Mild sexual violence*. ‘Has anybody against your will touched parts of your body other than genitals in a sexual way or forced you to touch parts of his or her body in a sexual way?’ ’Have you in any other way been sexually humiliated; e.g. by being forced to watch a porno movie or similar against your will, forced to show your body naked, or forced to watch when somebody else showed his/her body?’
2. *Moderate sexual violence*. ‘Has anybody against your will touched your genitals, used your body to satisfy him/herself, or forced you to touch anybody else’s genitals?’
3. *Severe sexual violence*. ‘Has anybody against your will put or tried to put his penis into your vagina, mouth, or rectum; put or tried to put an object or other parts of the body into your vagina, mouth, or rectum?’

For each of these questions the women could answer who the perpetrator was and if the violence was experienced during the past year. Women answering ‘yes’ to any of the questions regarding sexual violence were categorized as women with experiences of sexual violence.

*Intimate partner violence*. Furthermore, the participants answered questions about experiences of psychological violence (Have you experienced anybody systematically and for any longer period trying to repress, degrade, or humiliate you? Have you experienced anybody systematically and by threat or force trying to limit your contacts with others or totally control what you may or may not do? Have you experienced living in fear because somebody systematically and for a longer period has threatened you or somebody close to you?) or physical violence (Have you experienced anybody hitting you, smacking your face, or holding you firmly against your will? Have you experienced anybody hitting you with his/her fist(s) or with a hard object, kicking you, pushing you violently, giving you a beating, thrashing you, or doing anything similar to you? Have you experienced anybody threatening your life by, for instance, trying to strangle you, showing a weapon or a knife, or by any other similar act?). If the participants answered ‘yes’ to any of those questions and had a present or former partner as perpetrator they were categorized as women with experiences of intimate partner violence.

*Non-partner sexual violence*. Women who answered ‘yes’ to any of the questions regarding sexual violence and the perpetrator was other than a partner or former partner were categorized as women with experience of non-partner sexual violence. The women who answered ‘yes’ to any of the questions regarding sexual violence and the perpetrator was a partner or former partner were excluded from the ‘non-partner sexual violence’ group.

Data analysed in this study came from the questionnaire answers and the interviews. No additional information was collected from the women’s medical journals.

### Statistical analysis

SPSS version 24.0 was used for the statistical analyses. The prevalence of sexual violence among the participants was assessed. Comparisons were made between the women who confirmed experiences of sexual violence and those who did not, regarding known risk factors (i.e. age, educational level, occupation, and place of birth). Furthermore, lifetime prevalence of intimate partner violence was compared between women with and without experiences of non-partner sexual violence to examine associations between sexual violence from a non-partner and other forms of violence. Bivariate associations were examined using the chi-square test. Statistical significance was set at a value of *P* < 0.05.

The study was approved by the Regional Ethical Review Board in Uppsala University (Dnr 2005:219).

## Results

### Prevalence of sexual violence

A total of 1517 questionnaires were distributed to women seeking care at the family planning unit. Of those, 1286 (85%) women agreed to participate. Sixty women did not state their background information and were therefore excluded from the analyses. The age of the women ranged from 15 to 55 years. Among the participants (*n* = 1226), 326 (27%) answered ‘yes’ to any of the questions regarding experiences of sexual violence during the interview or in the questionnaire. During the interviews, 192 (16%) of the participants reported that they had been sexually abused, 19.2% (37/192) before they were 13 years of age, 40.1% (77/192) when they were 13–18 years of age, and 41.1% (79/192) when they were over 18 years of age. In the questionnaire, 310 (25%) of the women answered ‘yes’ to some or all four questions regarding experiences of mild, moderate, or severe sexual violence. Of those women, 157 (51%) reported experiences of severe sexual violence. Sexual violence during the past year was reported by 38 (3%) of the participants.

Of all the women who reported exposure to sexual violence in the questionnaire, 220 (71%) stated that the perpetrator was a non-partner.

### Experiences of sexual violence and background characteristics

It was more common that women with experiences of sexual violence had 9 or less years of education (*P* = 0.024), were students or without occupation (*P* = 0.037), and were not in a current relationship (*P* < 0.001) (Table 1). Women with no experiences of sexual violence were more likely to be in longer relationships. There was also a difference between the two groups, with the women with experiences of sexual violence having a higher total lifetime experience of psychological and/or physical violence from a present or former partner (*P* < 0.001).

**Table 1.** Distribution of study participants by lifetime sexual violence exposure, a series of background characteristics, and experience of emotional and/or physical violence from a partner or former partner.

Characteristics	Experience of sexual violence *n* (%)	No experience of sexual violence *n* (%)	Missing values	*P* value^a^
Current age (years)				0.156
15–19	42 (34)	83 (66)		
20–30	171 (26)	479 (74)		
>31	113 (25)	338 (75)		
Relationship			27	<0.001
Not in relationship	85 (35)	156 (65)		
Less than 1 year	69 (31)	159 (69)		
1–5 years	106 (26)	295 (74)		
Over 6 years	57 (18)	262 (82)		
Place of birth			22	0.131
Scandinavia	279 (26)	813 (74)		
Other than Scandinavia	36 (32)	76 (68)		
Education				0.024
≤9 years of school	64 (33)	129 (67)		
>9 years of school	262 (25)	771 (75)		
Occupation				0.037
Gainfully employed	159 (24)	512 (76)		
Student	113 (31)	255 (69)		
Without occupation^b^	54 (29)	133 (71)		
Experience of psychological and/or physical violence from a partner or former partner			10	<0.001
Yes	151 (47)	170 (53)		
No	179 (20)	716 (80)		

a Pearson’s chi-square test.

b Unemployed, parental leave, sick leave.

Of the women who reported experiences of sexual violence during the interviews or in the questionnaires, 186 (57%) were abused when they were younger than 18 years of age. In the questionnaire, 71 (23%) respondents stated that they had been subjected to severe penetrating sexual violence when they were younger than 18 years of age. In 55 of those cases, the violence was committed by a non-partner (data not presented).

### Experience of non-partner sexual violence and intimate partner violence

The women with experience of non-partner sexual violence were more likely to report experiences of psychological and physical violence from a present or former partner compared to women with no experiences of non-partner sexual violence (*P* < 0.001). There were no significant differences between the groups regarding experiences of intimate partner violence during the past year (Table 2).

**Table 2.** Distribution of participants by sexual violence perpetrator and type of intimate partner violence.

Type of intimate partner violence	*n*	Experience of non-partner sexual violence *n* (%)	No experience of non-partner sexual violence *n* (%)	*P* value^a^
Interviews				
Have you ever experienced physical or psychological violence from a present or former partner?	npSV: 220 no npSV: 909	58 (26)	138 (15)	<0.001
Have you during the past year been slapped, kicked, shoved, or in other ways harmed by a present or former partner?	npSV: 219 no npSV: 908	10 (5)	21 (2)	0.067
Questionnaire				
Experience of psychological violence from a partner or former partner	npSV: 218 no npSV: 909	67 (31)	121 (13)	<0.001
Experience of physical violence from a partner or former partner	npSV: 217 no npSV: 908	75 (24)	177 (13)	0.003
Total experience of intimate partner violence (interviews + questionnaire)	npSV: 220 no npSV: 911	69 (31)	281 (20)	<0.001

a Pearson’s chi-square test.

npSV = experiences of non-partner sexual violence.

## Discussion

Results of the present study indicate that the prevalence of different types of sexual violence is high within the context of a family planning unit in Sweden. Experiences of sexual violence were reported by 27% of the participants in this study. This rate can be compared to other prevalence studies in Sweden, the Nordic countries, and Europe. In a national survey from 2014 in Sweden with a representative sample of 5681 women, 42% of the women reported experiences of sexual violence (3). A study among Swedish adolescents aged 17–23 years old showed that the prevalence of sexual violence was 32% (20). A Nordic cross-country study revealed a prevalence of sexual violence of 24.1% of the 3641 respondents (19). In Europe, the prevalence of sexual violence has been estimated to an average of 11% among European Union countries (2). Sexual violence during the past year was reported by 3% of the women in this study. This is consistent with other studies where the 12-month prevalence of sexual violence was 2%–5.8% (2,3).

Among the participants who experienced sexual violence, 57% (15% of all the respondents in the study) specified that they were subjected to violence when they were younger than 18 years of age. The European survey showed an occurrence of sexual violence before 15 years of age of 12% of the respondents (2). This is in accordance to WHO global prevalence studies, including 133 countries, reporting 20% of women having been sexually abused as children (21).

The prevalence of non-partner sexual violence was 71% in this study. The respective prevalence has been estimated globally to 7.2% (1) and 6% in the European Union countries (2). In addition to acquaintance, friend, or unknown persons, etc., family members that commit sexual violence are also included as non-partner perpetrators. The higher prevalence of non-partner sexual violence in our study is in line with the large number of participants who reported exposure of sexual violence when they were younger than 18 years of age.

Women with experience of sexual violence were not as often in a relationship in comparison to women without sexual violence experiences, and the latter group was more likely to be in longer relationships. Considering known risk factors such as educational level and occupation, women with experiences of sexual violence reported lower educational level, were students or without occupation compared to women without experiences of sexual violence. Socioeconomic status was thus strongly associated with experiences of sexual violence in this setting in Sweden, in line with studies from other countries (22).

Our study results of a high prevalence of sexual violence among teenagers raise the question on the need for preventive efforts in this group. Of the women who confirmed experience of non-partner sexual violence in particular, 31% stated that they also had lifetime experience of psychological and/or physical violence from an intimate partner. This could be explained by the fact that the majority of the women in our study reported experience of sexual violence when they were younger than 18 years of age, and that one of the consequences of child sexual abuse is the risk of lifetime co-occurrence of violence (23,24). Our results are in line with findings from the Swedish national survey which showed a distinct association between experience of sexual violence before 18 years of age and exposure to violence as an adult (3).

Among the strengths of this study are the large number of participants and available information on the individual level of many related parameters, as well as the specification of perpetrator of sexual violence (partner versus non-partner). A limitation of the study could be the participants’ self-report on experience of different types of violence. Nevertheless, by asking specifically structured questions about violence, we minimized the risk of misinterpretations regarding the definition of violence. The study data were collected some years ago, but there have been no big legislative or policy changes during this period. Family planning units are structured in similar ways throughout Sweden, but using solely a family planning unit from one region as a recruitment area may have affected the degree of generalizability of the findings. Although all women wanting termination of pregnancy need to visit the family planning unit, the questionnaire being available only in Swedish probably resulted in failure to include a number of immigrants who did not speak Swedish. The impact of the exclusion of this group of women on the results is hard to hypothesize about, but based on the literature, associations present in this study have been reported in other cultures and thus are not expected to greatly differ in immigrant populations in Sweden (1).

Experiences of sexual violence were common among women participating in this study. Many of the respondents were young when they were exposed to violence, and lifetime co-occurrence of violence was common. Thus, the results of this study have important clinical implications. Identification of victims within the health care system would not only enable the development of effective interventions for treatment but also a possibility to prevent future violence.

## References

[1] World Health Organization Global and regional estimates of violence against women: prevalence and health effects of intimate partner violence and non-partner sexual violence. Geneva: World Health Organization; 2013.

[2] European Union Agency for Fundamental Rights Violence against women: an EU-wide survey. Main results. Luxemburg: Publications Office of the European Union; 2014.

[3] National Centre for Knowledge on Men’s Violence Against Women Violence and health: a national prevalence study on the exposure to violence of women and men and possible health implications. NCK-report; 2014 p. 1.

[4] Krug EG, Dahlberg LL, Mercy JA, Zwi AB, Lozano R, editors. World report on violence and health. Geneva: World Health Organization; 2002.

[5] Paras ML, Murad MH, Chen LP, Goranson EN, Sattler AL, Colbenson KM, et al. Sexual abuse and lifetime diagnosis of somatic disorders: a systematic review and meta-analysis. JAMA. 2009;302:550–61. 19654389 10.1001/jama.2009.1091

[6] Ellsberg M, Jansen H, Heise L, Watts CH, Garcia-Moreno C Intimate partner violence and women’s physical and mental health in the WHO multi-country study on women’s health and domestic violence: an observational study. Lancet. 2008;371:1165–72. 18395577 10.1016/S0140-6736(08)60522-X

[7] Williams MC, Clear RM, Coker LA Sexual coercion and sexual violence at first intercourse associated with sexually transmitted infections. Sex Transm Dis. 2013;40:771–5. 24275726 10.1097/OLQ.0000000000000011 PMC3927639

[8] Gisladottir A, Harlow BL, Gudmundsdottir B, Bjarnadottir RI, Jonsdottir E, Aspelund T, et al. Risk factors and health during pregnancy among women previously exposed to sexual violence. Acta Obstet Gynecol Scand. 2014;93:351–8. 24490826 10.1111/aogs.12331

[9] Jina R, Thomas SL Health consequences of sexual violence against women. Best Pract Res Clin Obstet Gynaecol. 2013;27:15–26. 22975432 10.1016/j.bpobgyn.2012.08.012

[10] Taylor S, Pugh J, Goodwach Coles J Sexual violence in women. The importance of identifying a history of sexual violence. Aust Fam Physician. 2012;41:638–41. 22762078

[11] Mason F, Lodrick Z Psychological consequences of sexual assault. Best Pract Res Clin Obstet Gynaecol 2013;27:27–37. 23182852 10.1016/j.bpobgyn.2012.08.015

[12] Chivers-Wilson K Sexual assault and posttraumatic stress disorder: a review of the biological, psychological and sociological factors and treatments. Mcgill J Med. 2006;9:111–18. 18523613 PMC2323517

[13] World Health Organization/London School of Hygiene and Tropical Medicine Preventing intimate partner violence and sexual violence against women: taking action and generating evidence. Geneva: World Health Organization; 2010.

[14] Simmons J, Wijma B, Swahnberg K Lifetime co-occurrence of violence victimization and symptoms of psychological ill health: a cross-sectional study of Swedish male and female clinical and population samples. BMC Public Health. 2015;15:1979. 10.1186/s12889-015-2311-3 PMC4587579 26415496

[15] Hamby S, Grych J The web of violence. Exploring connections among different forms of interpersonal violence and abuse. Springer briefs in sociology New York: Springer; 2013.

[16] Öberg M, Stenson K, Skalkidou A, Heimer G Prevalence of intimate partner violence among women seeking termination of pregnancy compared to women seeking contraceptive counseling. Acta Obstet Gynecol Scand. 2014;93:45–51. 24117134 10.1111/aogs.12279

[17] KL. Soeken The abuse assessment screen: a clinical instrument to measure frequency, severity and perpetrator of abuse against women In: Campbell J, editor. Empowering survivors of abuse: health care for battered women and their children. Newbury Park, CA: Sage; 1998 p. 195–203.

[18] Swahnberg K, Wijma B The Norvold Abuse Questionnaire (NorAQ): validation of new measures of emotional, physical and sexual abuse, and abuse in the health care system among women. Eur J Public Health. 2003;361:2107–13. 10.1093/eurpub/13.4.361 14703325

[19] Wijma B, Schei B, Swahnberg K, Hilden M, Offerdal K, Pikarinen U, et al. Emotional, physical and sexual abuse among patients visiting gynaecology clinics: a Nordic cross-sectional study. Lancet. 2003;361:2107–13. 12826432 10.1016/s0140-6736(03)13719-1

[20] Danielsson I, Blom H, Nilses C, Heimer G, Högberg U Gendered patterns of high violence exposure among Swedish youth. Acta Obstet Gynecol Scand. 2009;88:528–35. 19353335 10.1080/00016340902846056

[21] World Health Organization Global status report on violence prevention 2014 Geneva: World Health Organization; 2014.

[22] García-Moreno C, Stöckl H Protection of sexual and reproductive rights: addressing violence against women. Int J Gynaecol Obstet. 2009;106:144–7. 19560770 10.1016/j.ijgo.2009.03.053

[23] Simma C, Postmus LJ, Lee I Sexual revictimization in adult women: examining factors associated with their childhood and adulthood experiences. J Child Sex Abus. 2012;21:593–611. 22994695 10.1080/10538712.2012.690836

[24] de Haas S, van Berlo W, Bakker F, Vanwesenbeeck I Prevalence and characteristics of sexual violence in the Netherlands, the risk for revictimization and pregnancy: results from a national population’s survey. Violence Vict. 2012;27:592–608. 22978077 10.1891/0886-6708.27.4.592

